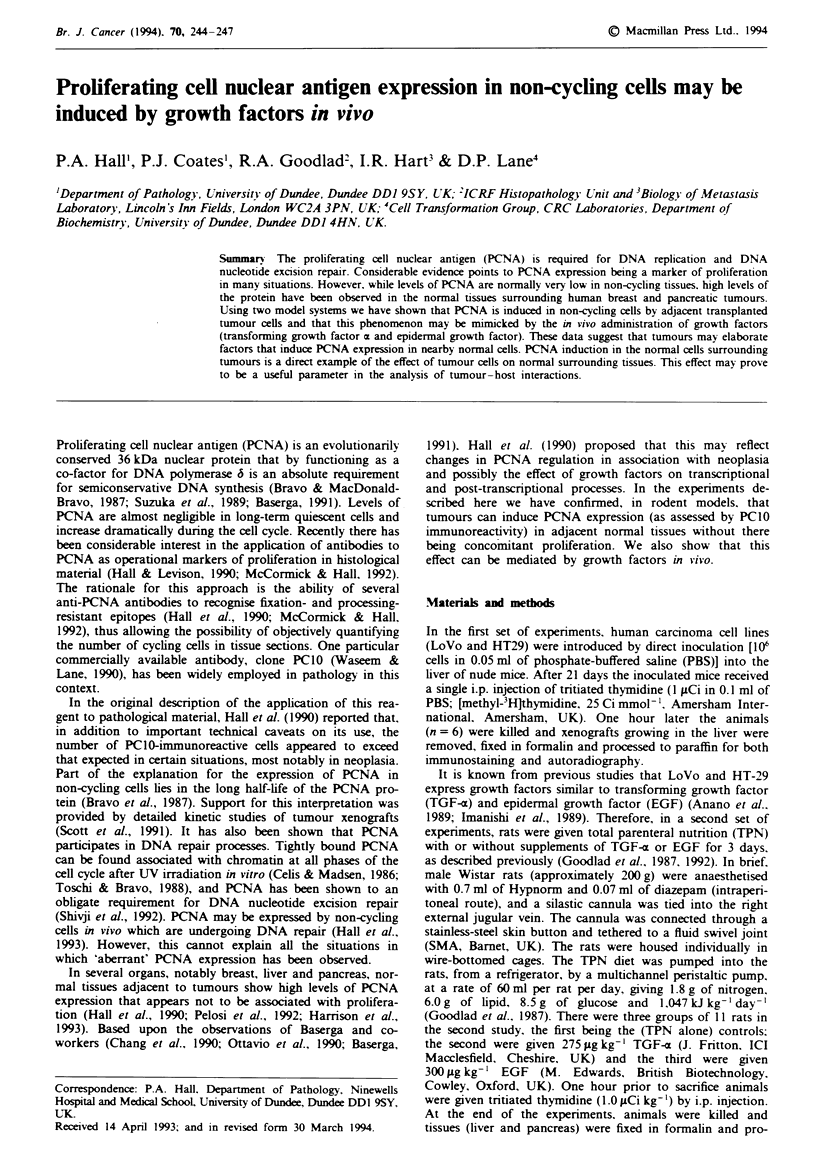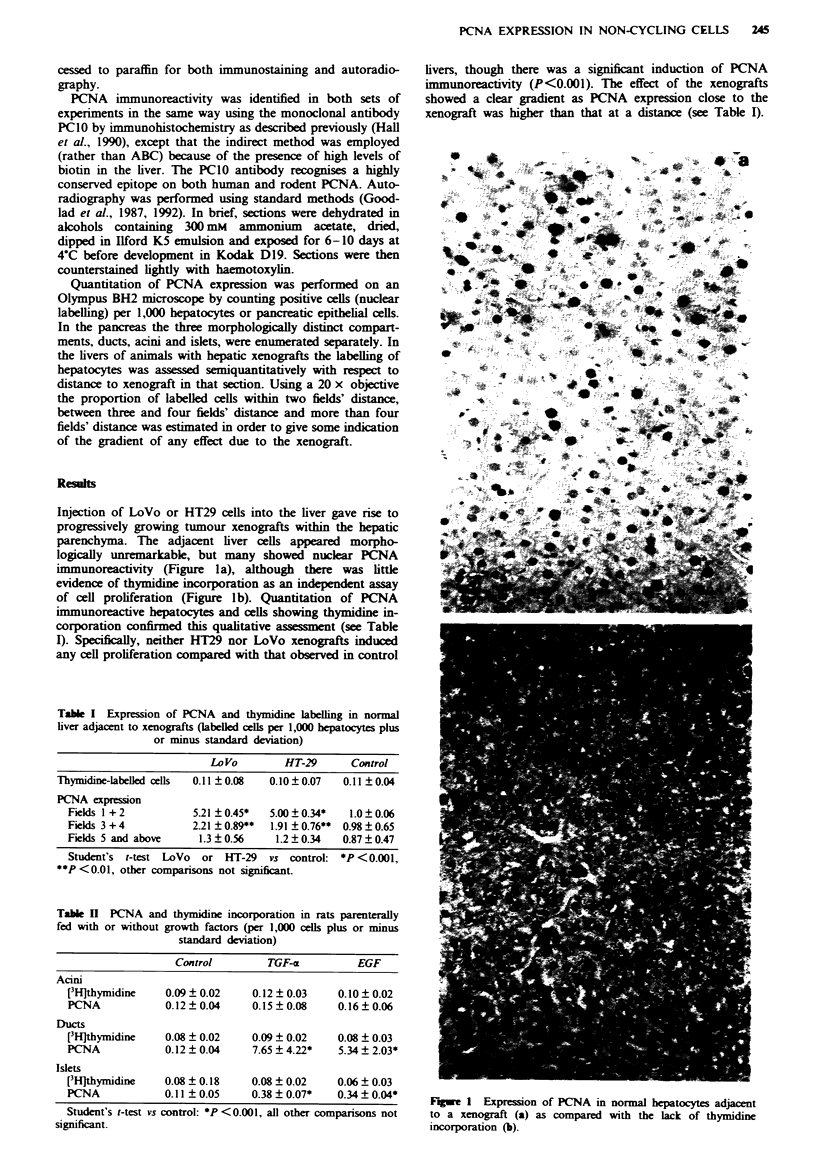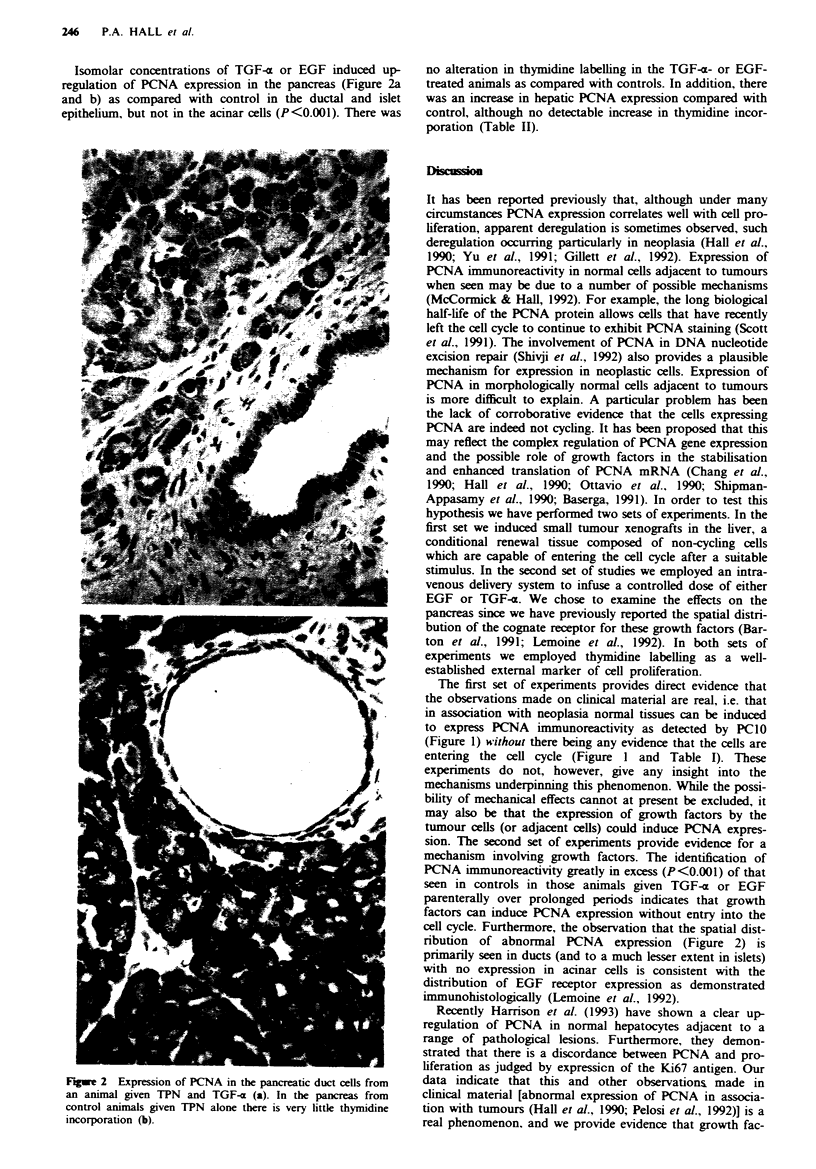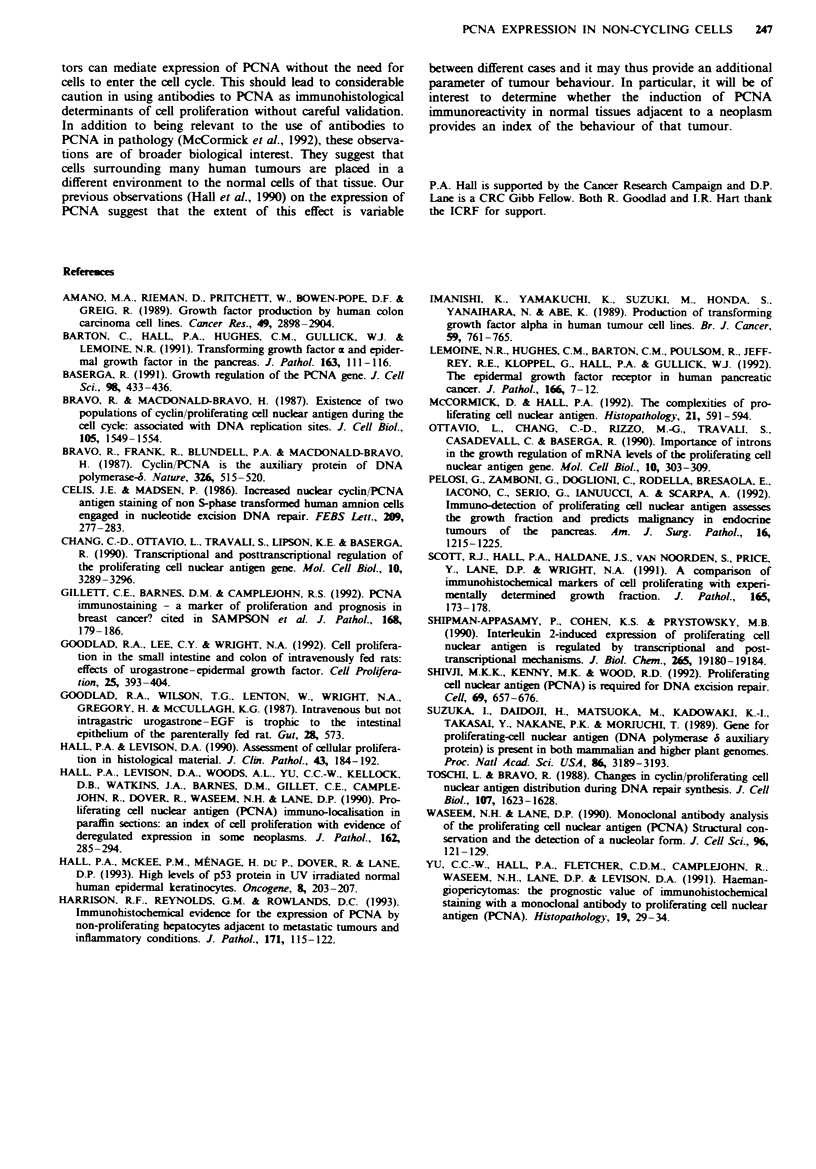# Proliferating cell nuclear antigen expression in non-cycling cells may be induced by growth factors in vivo.

**DOI:** 10.1038/bjc.1994.287

**Published:** 1994-08

**Authors:** P. A. Hall, P. J. Coates, R. A. Goodlad, I. R. Hart, D. P. lane

**Affiliations:** Department of Pathology, University of Dundee, UK.

## Abstract

**Images:**


Br. J. Cancer (1994), 70, 244-247 © Macmillan Press Ltd., 1994

### Proliferating cell nuclear antigen expression in non-cycling cells may be induced by growth factors *in vivo*

P.A. Hall[1], P.J. Coates[1], R.A. Goodlad[2], I.R. Hart[3] & D.P. Lane[4]

[1]*Department of Pathology, University of Dundee, Dundee DD1 9SY, UK;* [2]*ICRF Histopathology Unit and* [3]*Biology of Metastasis Laboratory, Lincoln's Inn Fields, London WC2A 3PN, UK;* [4]*Cell Transformation Group, CRC Laboratories, Department of Biochemistry, University of Dundee, Dundee DD1 4HN, UK.*

**Summary** The proliferating cell nuclear antigen (PCNA) is required for DNA replication and DNA nucleotide excision repair. Considerable evidence points to PCNA expression being a marker of proliferation in many situations. However, while levels of PCNA are normally very low in non-cycling tissues, high levels of the protein have been observed in the normal tissues surrounding human breast and pancreatic tumours. Using two model systems we have shown that PCNA is induced in non-cycling cells by adjacent transplanted tumour cells and that this phenomenon may be mimicked by the *in vivo* administration of growth factors (transforming growth factor α and epidermal growth factor). These data suggest that tumours may elaborate factors that induce PCNA expression in nearby normal cells. PCNA induction in the normal cells surrounding tumours is a direct example of the effect of tumour cells on normal surrounding tissues. This effect may prove to be a useful parameter in the analysis of tumour-host interactions.

Proliferating cell nuclear antigen (PCNA) is an evolutionarily conserved 36 kDa nuclear protein that by functioning as a co-factor for DNA polymerase δ is an absolute requirement for semiconservative DNA synthesis (Bravo & MacDonald-Bravo, 1987; Suzuka *et al.*, 1989; Baserga, 1991). Levels of PCNA are almost negligible in long-term quiescent cells and increase dramatically during the cell cycle. Recently there has been considerable interest in the application of antibodies to PCNA as operational markers of proliferation in histological material (Hall & Levison, 1990; McCormick & Hall, 1992). The rationale for this approach is the ability of several anti-PCNA antibodies to recognise fixation- and processing-resistant epitopes (Hall *et al.*, 1990; McCormick & Hall, 1992), thus allowing the possibility of objectively quantifying the number of cycling cells in tissue sections. One particular commercially available antibody, clone PC10 (Waseem & Lane, 1990), has been widely employed in pathology in this context.

In the original description of the application of this reagent to pathological material, Hall *et al.* (1990) reported that, in addition to important technical caveats on its use, the number of PC10-immunoreactive cells appeared to exceed that expected in certain situations, most notably in neoplasia. Part of the explanation for the expression of PCNA in non-cycling cells lies in the long half-life of the PCNA protein (Bravo *et al.*, 1987). Support for this interpretation was provided by detailed kinetic studies of tumour xenografts (Scott *et al.*, 1991). It has also been shown that PCNA participates in DNA repair processes. Tightly bound PCNA can be found associated with chromatin at all phases of the cell cycle after UV irradiation *in vitro* (Celis & Madsen, 1986; Toschi & Bravo, 1988), and PCNA has been shown to an obligate requirement for DNA nucleotide excision repair (Shivji *et al.*, 1992). PCNA may be expressed by non-cycling cells *in vivo* which are undergoing DNA repair (Hall *et al.*, 1993). However, this cannot explain all the situations in which 'aberrant' PCNA expression has been observed.

In several organs, notably breast, liver and pancreas, normal tissues adjacent to tumours show high levels of PCNA expression that appears not to be associated with proliferation (Hall *et al.*, 1990; Pelosi *et al.*, 1992; Harrison *et al.*, 1993). Based upon the observations of Baserga and co-workers (Chang *et al.*, 1990; Ottavio *et al.*, 1990; Baserga, 1991), Hall *et al.* (1990) proposed that this may reflect changes in PCNA regulation in association with neoplasia and possibly the effect of growth factors on transcriptional and post-transcriptional processes. In the experiments described here we have confirmed, in rodent models, that tumours can induce PCNA expression (as assessed by PC10 immunoreactivity) in adjacent normal tissues without there being concomitant proliferation. We also show that this effect can be mediated by growth factors *in vivo*.

### Materials and methods

In the first set of experiments, human carcinoma cell lines (LoVo and HT29) were introduced by direct inoculation [$10^6$ cells in 0.05 ml of phosphate-buffered saline (PBS)] into the liver of nude mice. After 21 days the inoculated mice received a single i.p. injection of tritiated thymidine (1 μCi in 0.1 ml of PBS; [methyl-$^3$H]thymidine, 25 Ci $mmol^{-1}$, Amersham International, Amersham, UK). One hour later the animals ($n = 6$) were killed and xenografts growing in the liver were removed, fixed in formalin and processed to paraffin for both immunostaining and autoradiography.

It is known from previous studies that LoVo and HT-29 express growth factors similar to transforming growth factor (TGF-α) and epidermal growth factor (EGF) (Anano *et al.*, 1989; Imanishi *et al.*, 1989). Therefore, in a second set of experiments, rats were given total parenteral nutrition (TPN) with or without supplements of TGF-α or EGF for 3 days, as described previously (Goodlad *et al.*, 1987, 1992). In brief, male Wistar rats (approximately 200 g) were anaesthetised with 0.7 ml of Hypnorm and 0.07 ml of diazepam (intraperitoneal route), and a silastic cannula was tied into the right external jugular vein. The cannula was connected through a stainless-steel skin button and tethered to a fluid swivel joint (SMA, Barnet, UK). The rats were housed individually in wire-bottomed cages. The TPN diet was pumped into the rats, from a refrigerator, by a multichannel peristaltic pump, at a rate of 60 ml per rat per day, giving 1.8 g of nitrogen, 6.0 g of lipid, 8.5 g of glucose and 1,047 kJ $kg^{-1}$ $day^{-1}$ (Goodlad *et al.*, 1987). There were three groups of 11 rats in the second study, the first being the (TPN alone) controls; the second were given 275 μg $kg^{-1}$ TGF-α (J. Fritton, ICI Macclesfield, Cheshire, UK) and the third were given 300 μg $kg^{-1}$ EGF (M. Edwards, British Biotechnology, Cowley, Oxford, UK). One hour prior to sacrifice animals were given tritiated thymidine (1.0 μCi $kg^{-1}$) by i.p. injection. At the end of the experiments, animals were killed and tissues (liver and pancreas) were fixed in formalin and pro-

Correspondence: P.A. Hall, Department of Pathology, Ninewells Hospital and Medical School, University of Dundee, Dundee DD1 9SY, UK.
Received 14 April 1993; and in revised form 30 March 1994.

cessed to paraffin for both immunostaining and autoradiography.

PCNA immunoreactivity was identified in both sets of experiments in the same way using the monoclonal antibody PC10 by immunohistochemistry as described previously (Hall *et al*., 1990), except that the indirect method was employed (rather than ABC) because of the presence of high levels of biotin in the liver. The PC10 antibody recognises a highly conserved epitope on both human and rodent PCNA. Autoradiography was performed using standard methods (Goodlad *et al*., 1987, 1992). In brief, sections were dehydrated in alcohols containing 300 mM ammonium acetate, dried, dipped in Ilford K5 emulsion and exposed for 6–10 days at 4°C before development in Kodak D19. Sections were then counterstained lightly with haemotoxylin.

Quantitation of PCNA expression was performed on an Olympus BH2 microscope by counting positive cells (nuclear labelling) per 1,000 hepatocytes or pancreatic epithelial cells. In the pancreas the three morphologically distinct compartments, ducts, acini and islets, were enumerated separately. In the livers of animals with hepatic xenografts the labelling of hepatocytes was assessed semiquantitatively with respect to distance to xenograft in that section. Using a 20 × objective the proportion of labelled cells within two fields' distance, between three and four fields' distance and more than four fields' distance was estimated in order to give some indication of the gradient of any effect due to the xenograft.

### Results

Injection of LoVo or HT29 cells into the liver gave rise to progressively growing tumour xenografts within the hepatic parenchyma. The adjacent liver cells appeared morphologically unremarkable, but many showed nuclear PCNA immunoreactivity (Figure 1a), although there was little evidence of thymidine incorporation as an independent assay of cell proliferation (Figure 1b). Quantitation of PCNA immunoreactive hepatocytes and cells showing thymidine incorporation confirmed this qualitative assessment (see Table I). Specifically, neither HT29 nor LoVo xenografts induced any cell proliferation compared with that observed in control livers, though there was a significant induction of PCNA immunoreactivity ($P<0.001$). The effect of the xenografts showed a clear gradient as PCNA expression close to the xenograft was higher than that at a distance (see Table I).

**Table I** Expression of PCNA and thymidine labelling in normal liver adjacent to xenografts (labelled cells per 1,000 hepatocytes plus or minus standard deviation)

| | *LoVo* | *HT-29* | *Control* |
|---|---|---|---|
| Thymidine-labelled cells | 0.11 ± 0.08 | 0.10 ± 0.07 | 0.11 ± 0.04 |
| PCNA expression | | | |
| Fields 1 + 2 | 5.21 ± 0.45* | 5.00 ± 0.34* | 1.0 ± 0.06 |
| Fields 3 + 4 | 2.21 ± 0.89** | 1.91 ± 0.76** | 0.98 ± 0.65 |
| Fields 5 and above | 1.3 ± 0.56 | 1.2 ± 0.34 | 0.87 ± 0.47 |

Student's *t*-test LoVo or HT-29 *vs* control: $^{*}P<0.001$, $^{**}P<0.01$, other comparisons not significant.

**Table II** PCNA and thymidine incorporation in rats parenterally fed with or without growth factors (per 1,000 cells plus or minus standard deviation)

| | *Control* | *TGF-α* | *EGF* |
|---|---|---|---|
| Acini | | | |
| [$^{3}$H]thymidine | 0.09 ± 0.02 | 0.12 ± 0.03 | 0.10 ± 0.02 |
| PCNA | 0.12 ± 0.04 | 0.15 ± 0.08 | 0.16 ± 0.06 |
| Ducts | | | |
| [$^{3}$H]thymidine | 0.08 ± 0.02 | 0.09 ± 0.02 | 0.08 ± 0.03 |
| PCNA | 0.12 ± 0.04 | 7.65 ± 4.22* | 5.34 ± 2.03* |
| Islets | | | |
| [$^{3}$H]thymidine | 0.08 ± 0.18 | 0.08 ± 0.02 | 0.06 ± 0.03 |
| PCNA | 0.11 ± 0.05 | 0.38 ± 0.07* | 0.34 ± 0.04* |

Student's *t*-test *vs* control: $^{*}P<0.001$, all other comparisons not significant.

**Figure 1** Expression of PCNA in normal hepatocytes adjacent to a xenograft (**a**) as compared with the lack of thymidine incorporation (**b**).

Isomolar concentrations of TGF-α or EGF induced up-regulation of PCNA expression in the pancreas (Figure 2a and b) as compared with control in the ductal and islet epithelium, but not in the acinar cells ($P<0.001$). There was no alteration in thymidine labelling in the TGF-α- or EGF-treated animals as compared with controls. In addition, there was an increase in hepatic PCNA expression compared with control, although no detectable increase in thymidine incorporation (Table II).

**Figure 2** Expression of PCNA in the pancreatic duct cells from an animal given TPN and TGF-α (**a**). In the pancreas from control animals given TPN alone there is very little thymidine incorporation (**b**).

### Discussion

It has been reported previously that, although under many circumstances PCNA expression correlates well with cell proliferation, apparent deregulation is sometimes observed, such deregulation occurring particularly in neoplasia (Hall *et al.*, 1990; Yu *et al.*, 1991; Gillett *et al.*, 1992). Expression of PCNA immunoreactivity in normal cells adjacent to tumours when seen may be due to a number of possible mechanisms (McCormick & Hall, 1992). For example, the long biological half-life of the PCNA protein allows cells that have recently left the cell cycle to continue to exhibit PCNA staining (Scott *et al.*, 1991). The involvement of PCNA in DNA nucleotide excision repair (Shivji *et al.*, 1992) also provides a plausible mechanism for expression in neoplastic cells. Expression of PCNA in morphologically normal cells adjacent to tumours is more difficult to explain. A particular problem has been the lack of corroborative evidence that the cells expressing PCNA are indeed not cycling. It has been proposed that this may reflect the complex regulation of PCNA gene expression and the possible role of growth factors in the stabilisation and enhanced translation of PCNA mRNA (Chang *et al.*, 1990; Hall *et al.*, 1990; Ottavio *et al.*, 1990; Shipman-Appasamy *et al.*, 1990; Baserga, 1991). In order to test this hypothesis we have performed two sets of experiments. In the first set we induced small tumour xenografts in the liver, a conditional renewal tissue composed of non-cycling cells which are capable of entering the cell cycle after a suitable stimulus. In the second set of studies we employed an intravenous delivery system to infuse a controlled dose of either EGF or TGF-α. We chose to examine the effects on the pancreas since we have previously reported the spatial distribution of the cognate receptor for these growth factors (Barton *et al.*, 1991; Lemoine *et al.*, 1992). In both sets of experiments we employed thymidine labelling as a well-established external marker of cell proliferation.

The first set of experiments provides direct evidence that the observations made on clinical material are real, i.e. that in association with neoplasia normal tissues can be induced to express PCNA immunoreactivity as detected by PC10 (Figure 1) *without* there being any evidence that the cells are entering the cell cycle (Figure 1 and Table I). These experiments do not, however, give any insight into the mechanisms underpinning this phenomenon. While the possibility of mechanical effects cannot at present be excluded, it may also be that the expression of growth factors by the tumour cells (or adjacent cells) could induce PCNA expression. The second set of experiments provide evidence for a mechanism involving growth factors. The identification of PCNA immunoreactivity greatly in excess ($P<0.001$) of that seen in controls in those animals given TGF-α or EGF parenterally over prolonged periods indicates that growth factors can induce PCNA expression without entry into the cell cycle. Furthermore, the observation that the spatial distribution of abnormal PCNA expression (Figure 2) is primarily seen in ducts (and to a much lesser extent in islets) with no expression in acinar cells is consistent with the distribution of EGF receptor expression as demonstrated immunohistologically (Lemoine *et al.*, 1992).

Recently Harrison *et al.* (1993) have shown a clear up-regulation of PCNA in normal hepatocytes adjacent to a range of pathological lesions. Furthermore, they demonstrated that there is a discordance between PCNA and proliferation as judged by expression of the Ki67 antigen. Our data indicate that this and other observations made in clinical material [abnormal expression of PCNA in association with tumours (Hall *et al.*, 1990; Pelosi *et al.*, 1992)] is a real phenomenon, and we provide evidence that growth factors can mediate expression of PCNA without the need for cells to enter the cell cycle. This should lead to considerable caution in using antibodies to PCNA as immunohistological determinants of cell proliferation without careful validation. In addition to being relevant to the use of antibodies to PCNA in pathology (McCormick *et al.*, 1992), these observations are of broader biological interest. They suggest that cells surrounding many human tumours are placed in a different environment to the normal cells of that tissue. Our previous observations (Hall *et al.*, 1990) on the expression of PCNA suggest that the extent of this effect is variable between different cases and it may thus provide an additional parameter of tumour behaviour. In particular, it will be of interest to determine whether the induction of PCNA immunoreactivity in normal tissues adjacent to a neoplasm provides an index of the behaviour of that tumour.

P.A. Hall is supported by the Cancer Research Campaign and D.P. Lane is a CRC Gibb Fellow. Both R. Goodlad and I.R. Hart thank the ICRF for support.

### References

AMANO, M.A., RIEMAN, D., PRITCHETT, W., BOWEN-POPE, D.F. & GREIG, R. (1989). Growth factor production by human colon carcinoma cell lines. *Cancer Res.*, **49**, 2898–2904.

BARTON, C., HALL, P.A., HUGHES, C.M., GULLICK, W.J. & LEMOINE, N.R. (1991). Transforming growth factor α and epidermal growth factor in the pancreas. *J. Pathol.* **163**, 111–116.

BASERGA, R. (1991). Growth regulation of the PCNA gene. *J. Cell Sci.*, **98**, 433–436.

BRAVO, R. & MACDONALD-BRAVO, H. (1987). Existence of two populations of cyclin/proliferating cell nuclear antigen during the cell cycle: associated with DNA replication sites. *J. Cell Biol.*, **105**, 1549–1554.

BRAVO, R., FRANK, R., BLUNDELL, P.A. & MACDONALD-BRAVO, H. (1987). Cyclin/PCNA is the auxiliary protein of DNA polymerase-δ. *Nature*, **326**, 515–520.

CELIS, J.E. & MADSEN, P. (1986). Increased nuclear cyclin/PCNA antigen staining of non S-phase transformed human amnion cells engaged in nucleotide excision DNA repair. *FEBS Lett.*, **209**, 277–283.

CHANG, C.-D., OTTAVIO, L., TRAVALI, S., LIPSON, K.E. & BASERGA, R. (1990). Transcriptional and posttranscriptional regulation of the proliferating cell nuclear antigen gene. *Mol. Cell Biol.*, **10**, 3289–3296.

GILLETT, C.E., BARNES, D.M. & CAMPLEJOHN, R.S. (1992). PCNA immunostaining – a marker of proliferation and prognosis in breast cancer? cited in SAMPSON *et al. J. Pathol.*, **168**, 179–186.

GOODLAD, R.A., LEE, C.Y. & WRIGHT, N.A. (1992). Cell proliferation in the small intestine and colon of intravenously fed rats: effects of urogastrone–epidermal growth factor. *Cell Proliferation*, **25**, 393–404.

GOODLAD, R.A., WILSON, T.G., LENTON, W., WRIGHT, N.A., GREGORY, H. & MCCULLAGH, K.G. (1987). Intravenous but not intragastric urogastrone–EGF is trophic to the intestinal epithelium of the parenterally fed rat. *Gut*, **28**, 573.

HALL, P.A. & LEVISON, D.A. (1990). Assessment of cellular proliferation in histological material. *J. Clin. Pathol.*, **43**, 184–192.

HALL, P.A., LEVISON, D.A., WOODS, A.L., YU, C.C.-W., KELLOCK, D.B., WATKINS, J.A., BARNES, D.M., GILLET, C.E., CAMPLEJOHN, R., DOVER, R., WASEEM, N.H. & LANE, D.P. (1990). Proliferating cell nuclear antigen (PCNA) immuno-localisation in paraffin sections: an index of cell proliferation with evidence of deregulated expression in some neoplasms. *J. Pathol.*, **162**, 285–294.

HALL, P.A., MCKEE, P.M., MÉNAGE, H. DU P., DOVER, R. & LANE, D.P. (1993). High levels of p53 protein in UV irradiated normal human epidermal keratinocytes. *Oncogene*, **8**, 203–207.

HARRISON, R.F., REYNOLDS, G.M. & ROWLANDS, D.C. (1993). Immunohistochemical evidence for the expression of PCNA by non-proliferating hepatocytes adjacent to metastatic tumours and inflammatory conditions. *J. Pathol.*, **171**, 115–122.

IMANISHI, K., YAMAKUCHI, K., SUZUKI, M., HONDA, S., YANAIHARA, N. & ABE, K. (1989). Production of transforming growth factor alpha in human tumour cell lines. *Br. J. Cancer*, **59**, 761–765.

LEMOINE, N.R., HUGHES, C.M., BARTON, C.M., POULSOM, R., JEFFREY, R.E., KLOPPEL, G., HALL, P.A. & GULLICK, W.J. (1992). The epidermal growth factor receptor in human pancreatic cancer. *J. Pathol.*, **166**, 7–12.

MCCORMICK, D. & HALL, P.A. (1992). The complexities of proliferating cell nuclear antigen. *Histopathology*, **21**, 591–594.

OTTAVIO, L., CHANG, C.-D., RIZZO, M.-G., TRAVALI, S., CASADEVALL, C. & BASERGA, R. (1990). Importance of introns in the growth regulation of mRNA levels of the proliferating cell nuclear antigen gene. *Mol. Cell Biol.*, **10**, 303–309.

PELOSI, G., ZAMBONI, G., DOGLIONI, C., RODELLA, BRESAOLA, E., IACONO, C., SERIO, G., IANUUCCI, A. & SCARPA, A. (1992). Immuno-detection of proliferating cell nuclear antigen assesses the growth fraction and predicts malignancy in endocrine tumours of the pancreas. *Am. J. Surg. Pathol.*, **16**, 1215–1225.

SCOTT, R.J., HALL, P.A., HALDANE, J.S., VAN NOORDEN, S., PRICE, Y., LANE, D.P. & WRIGHT, N.A. (1991). A comparison of immunohistochemical markers of cell proliferating with experimentally determined growth fraction. *J. Pathol.*, **165**, 173–178.

SHIPMAN-APPASAMY, P., COHEN, K.S. & PRYSTOWSKY, M.B. (1990). Interleukin 2-induced expression of proliferating cell nuclear antigen is regulated by transcriptional and post-transcriptional mechanisms. *J. Biol. Chem.*, **265**, 19180–19184.

SHIVJI, M.K.K., KENNY, M.K. & WOOD, R.D. (1992). Proliferating cell nuclear antigen (PCNA) is required for DNA excision repair. *Cell*, **69**, 657–676.

SUZUKA, I., DAIDOJI, H., MATSUOKA, M., KADOWAKI, K.-I., TAKASAI, Y., NAKANE, P.K. & MORIUCHI, T. (1989). Gene for proliferating-cell nuclear antigen (DNA polymerase δ auxiliary protein) is present in both mammalian and higher plant genomes. *Proc. Natl Acad. Sci. USA*, **86**, 3189–3193.

TOSCHI, L. & BRAVO, R. (1988). Changes in cyclin/proliferating cell nuclear antigen distribution during DNA repair synthesis. *J. Cell Biol.*, **107**, 1623–1628.

WASEEM, N.H. & LANE, D.P. (1990). Monoclonal antibody analysis of the proliferating cell nuclear antigen (PCNA) Structural conservation and the detection of a nucleolar form. *J. Cell Sci.*, **96**, 121–129.

YU, C.C.-W., HALL, P.A., FLETCHER, C.D.M., CAMPLEJOHN, R., WASEEM, N.H., LANE, D.P. & LEVISON, D.A. (1991). Haemangiopericytomas: the prognostic value of immunohistochemical staining with a monoclonal antibody to proliferating cell nuclear antigen (PCNA). *Histopathology*, **19**, 29–34.